# T-ScEmo4ALL Plus+: Treatment for social cognition disorders in patients with acquired brain injury and neuropsychiatric problems – A single case experimental design (SCED) study protocol

**DOI:** 10.1371/journal.pone.0358008

**Published:** 2026-09-18

**Authors:** Bente Raafs, Marjon Westerhof-Evers, Caroline van Heugten, Joke Spikman, Sophie Rijnen

**Affiliations:** 1 Center of Excellence for Brain Injury and Neuropsychiatry, GGZ Oost Brabant, Boekel, The Netherlands; 2 Faculty of Psychology and Neuroscience, Maastricht University, Maastricht, The Netherlands; 3 Department of Neurology, Clinical Neuropsychology, University of Groningen, University Medical Center Groningen, Groningen, The Netherlands; Public Library of Science, UNITED STATES OF AMERICA

## Abstract

**Introduction:**

Many patients with acquired brain injury (ABI) suffer from impaired social cognition, which may result in reduced social participation, quality of life and quality and stability of partner relationships. Social cognition problems often co-occur with, and in some cases contribute to, neuropsychiatric symptoms such as emotional dysregulation, disinhibition, and irritability, which may further complicate social functioning. Effective interventions targeting social cognition are therefore of particular importance for ABI patients with comorbid neuropsychiatric symptoms. The Treatment Social cognition and Emotion regulation (T-ScEmo) has shown promising effects on social cognition and emotion regulation in patients with traumatic brain injury (TBI), but has not yet been studied in ABI patients with comorbid neuropsychiatric problems. Therefore, the main aim of the current study is to examine the effect of T-ScEmo on social functioning in patients with ABI and comorbid neuropsychiatric problems.

**Methods and analysis:**

A non-concurrent, randomized, multiple-baseline single-case experimental design (SCED) will be employed to investigate the effectiveness of T-ScEmo in this study. At least 8 participants and a significant other will be randomly assigned to one of three baseline lengths. The primary outcome measures (social functioning, neuropsychiatric problems, relationship quality) will be assessed daily during baseline and twice a week during the treatment and maintenance phases. Secondary outcomes will be assessed at T0 (pre-treatment), T1 (post-treatment), and T2 (1-year follow-up). Both patients and the significant other will complete all outcome measures.

**Discussion:**

T-ScEmo may offer a valuable addition to neurorehabilitation practices for this specific target group of patients. Forthcoming results may contribute to more personalized and effective treatment strategies for the growing number of patients with ABI and comorbid neuropsychiatric problems.

**Trial registration:**

The study is registered in the database of clinicaltrials.gov (Treatment for Social Cognition Disorders (T-ScEmo) in Patients With Acquired Brain Injury and Comorbid Neuropsychiatric Problems | ClinicalTrials.gov) under identifier NCT06923683. Date registered: 04/17/2025.

## Introduction

Social cognition is a fundamental aspect of human behavior, encompassing the ability to attend to, process and interpret social information to understand other people, and react to their expectations or needs [1]. Social cognition is a complex, multifaceted construct that is challenging to define concisely. It can generally be described as “the ability to function appropriately in social and interpersonal situations” [2]. More specifically, social cognition consists of three interrelated aspects in which individuals with acquired brain injury (ABI) frequently experience difficulties [3,4]. First, perception of socially relevant information (e.g., reading facial expressions, body language, and emotional cues). Second, understanding and interpreting others’ thoughts and feelings (e.g., empathy, theory of mind (ToM), mentalization). Third, responding to social information (e.g., appropriately adjusting behavior to the situation) [1,5]. To address the complexity of social cognition, the Hierarchical Interdependent Taxonomy of Social cognition (HITS) model was developed. This biopsychosocial framework integrates expert consensus with empirical evidence and highlights the interdependence of key social cognitive processes. It offers a structured, theory-driven basis for assessment and intervention in both research and clinical settings [6].

Problems in social cognition are prevalent across a wide range of neurological, neuropsychiatric and developmental brain disorders, including traumatic brain injury (TBI), stroke, brain tumors, and autism spectrum disorders [7–9]. In ABI, especially when the orbitofrontal and ventromedial prefrontal cortices are affected, social cognition impairments are common [10,11]. Prevalence estimates suggest that 13%−47% of patients with ABI show difficulties in domains such as theory of mind, empathy, and facial emotion recognition [12,13]. However, these percentages likely underestimate the true prevalence, as social cognition is often not considered in routine neuropsychological assessments [14].

Social cognition problems represent a major source of burden for individuals with ABI and their families. Increasing evidence suggests that such problems contribute to socially inappropriate or disruptive behavior (e.g., disinhibition, lack of empathy, apathy) [15,16], which is often viewed as problematic by relatives [17]. These problems negatively impact the quality and stability of partner relationships, social participation, return to work, and quality of life [17–22]. Thus, given the central role of social cognition in everyday functioning, the treatment of deficits in social cognition following ABI is of critical importance [14].

Although the number of treatment studies targeting social cognition is growing, existing studies still face important limitations. Most studies focused on a single aspect of social cognition, included small sample sizes, lacked (long-term) follow-up data, and/or showed limited generalization to everyday-life social behavior [23–29]. In response, Westerhof-Evers et al. [5] developed a comprehensive, multifaceted intervention called T-ScEmo, targeting impairments in social cognition and emotion regulation. A randomized controlled trial (RCT; N = 61) demonstrated its effectiveness in improving facial affect recognition, ToM, empathic behavior, and societal participation of patients with TBI. Unlike most interventions, T-ScEmo addresses three core aspects of social cognition, includes involvement of significant others, and strongly emphasizes generalization to daily life. As a result, T-ScEmo is now established as an evidence-based treatment for social cognition impairments in individuals with TBI in the Netherlands.

Social cognition problems often co-occur with, and in some cases contribute to, neuropsychiatric problems after ABI. Patients with comorbid neuropsychiatric problems are however often excluded from treatment studies on social cognition. In this study, neuropsychiatric problems are broadly defined to include a wide spectrum of behavioral problems — such as disinhibition and aggression — as well as formal DSM-5 diagnoses including anxiety disorders, depression, and personality disorders. Neuropsychiatric behavioral problems, such as disinhibition, alexithymia, and aggression, frequently overlap with and/or result from underlying social cognition impairments [30,31]. Yet, neuropsychiatric symptoms are not exclusively attributable to social cognition deficits; many patients exhibit neuropsychiatric disturbances that arise independently of social cognitive impairments, reflecting a multifactorial pathophysiology [32]. In this study, patients present with neuropsychiatric problems of such severity and complexity that treatment within a specialized neuropsychiatric setting is indicated. These patients differ from those commonly seen in standard rehabilitation settings, who may also present with social cognition deficits, but exhibit less severe and disruptive neuropsychiatric symptoms.

A recent literature review by Torregrossa and colleagues [33] reported that 25% to 88% of individuals with moderate to severe ABI experience neuropsychiatric problems, including aggression, disinhibition, irritability, alexithymia, depression, anxiety, PTSD, or suicidality [33]. Moreover, a retrospective study found that 72.5% to 81.8% of patients with ABI and neuropsychiatric problems score low or very low on emotion recognition, with significantly lower scores in other domains of social cognition (e.g., empathy, ToM) compared to patients with ABI without such comorbidities [34]. Despite the high prevalence and severity of these problems, patients with ABI and comorbid neuropsychiatric problems are often excluded from treatment studies on social cognition. Only a few studies have evaluated interventions in this subgroup: two studies found positive effects of group-based treatments on social communication skills in patients with ABI and comorbidities, but only focused on social communication skills [35], and one RCT demonstrated the effectiveness of a treatment in this population, but included a small sample (N = 43) and lacked follow-up data [27]. This limited evidence is striking, especially given that social cognitive problems may be more pronounced and persistent in patients with ABI and comorbid neuropsychiatric problems [34].

The exclusion of patients with ABI and neuropsychiatric comorbidities in ABI research [5,21,25,28] is often justified on ethical or methodological grounds – such as concerns about decisional capacity or confounding effects on outcome measures. However, these exclusions limit the external validity and clinical applicability of research findings. For example, a systematic scoping review of 428 inpatient stroke rehabilitation RCTs found that 83% excluded patients with comorbidities, 54% excluded those with cognitive impairment, and 36% excluded those with prior stroke [36]. These exclusions risk creating a research-practice gap, whereby interventions validated in highly selective and homogeneous populations may not be applicable to real-world clinical settings.

Although the effectiveness of T-ScEmo has been demonstrated in individuals with TBI, it remains unknown whether the treatment is also effective in individuals with ABI and comorbid neuropsychiatric problems. Therefore, the primary aim is to examine the effect of T-ScEmo on social functioning in patients with ABI and comorbid neuropsychiatric problems (RQ1). Secondary aims include examining the effects of T-ScEmo on neuropsychiatric problems and quality of partner relationships in this population (RQ2).

### Hypotheses

#### Primary research question (RQ1).

Based on previous research in patients with TBI [5,21], we hypothesize that T-ScEmo will reduce social cognition problems in patients with ABI and comorbid neuropsychiatric problems. While T-ScEmo is a compensatory strategy training and thus not expected to directly improve underlying cognitive mechanisms, improvements in social behavior are anticipated due to skill acquisition, practice in daily life, and involvement of significant others. However, as the first modules specifically targets facial emotion recognition, we also expect improvements on the FEEST (i.e., emotion recognition).

We also expect improvements in social communication and interaction, as the treatment targets emotion recognition, ToM, perspective taking, awareness, inhibition of undesired social behavior and socially desired behavior. Improved compensation for deficits is expected to enhance participation in social contexts.

#### Secondary research questions (RQ2).

In addition, we hypothesize that T-ScEmo will reduce neuropsychiatric problems in patients with ABI and comorbid neuropsychiatric problems. Social cognition problems often co-occur with, and in some cases contribute to, with neuropsychiatric symptoms following acquired brain injury (ABI). These social cognition difficulties are also commonly observed in other psychiatric disorders such as autism spectrum disorders and schizophrenia [8,37]. Although our study focuses on patients with ABI, improvements in social cognition have the potential to alleviate distress and behavioral dysregulation associated with neuropsychiatric symptoms across different conditions [28].

Finally, we hypothesize that the quality of partner relationships will improve. Involving a partner in the treatment may increase mutual understanding and support, while better social responsiveness on the part of the patient can positively influence relationship quality.

## Methods

### Study design

#### Phases and measurements.

**Fig 1:**
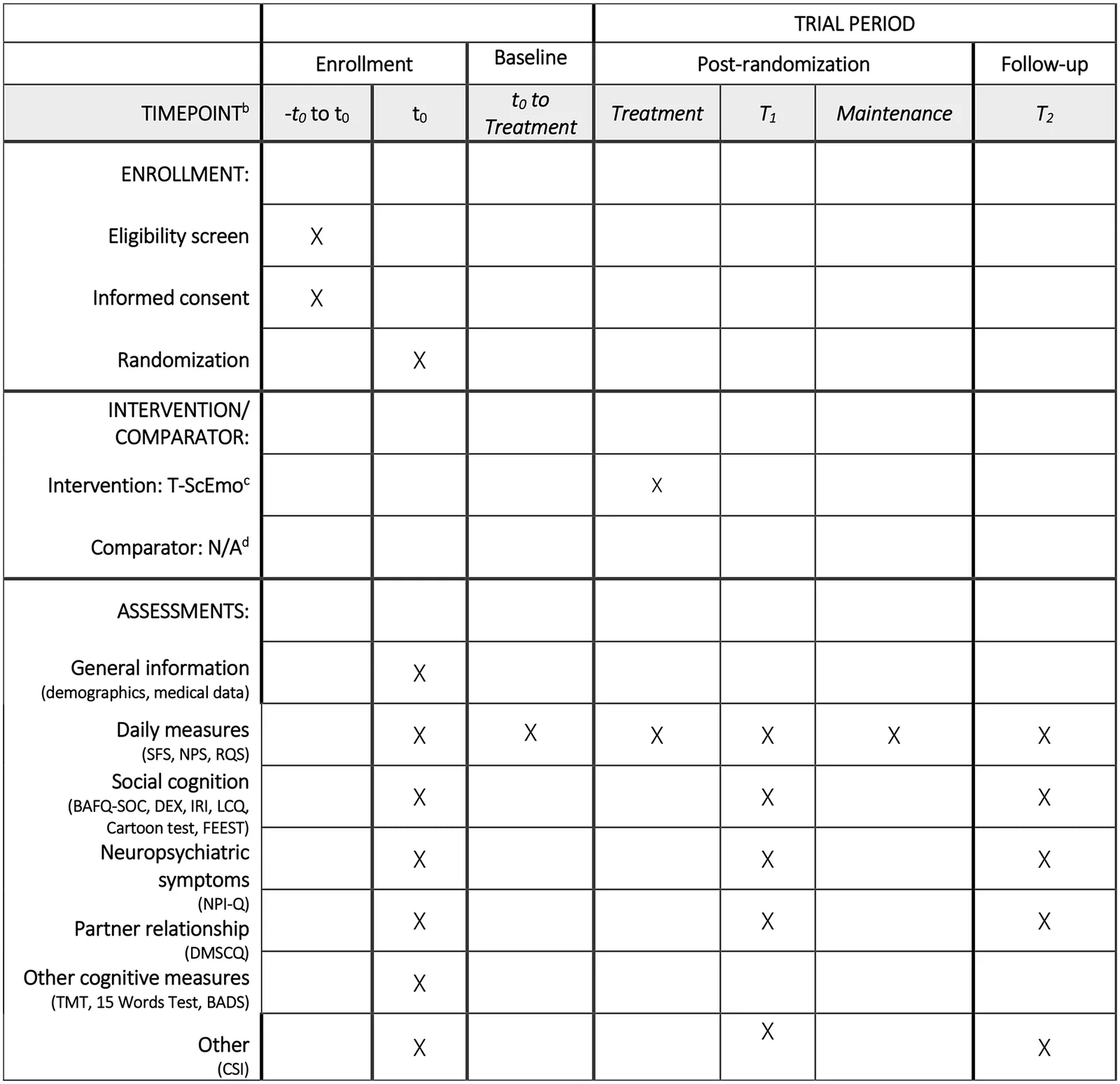
Participant timeline: Schedule of enrollment, interventions, and assessments.

A non-concurrent, randomized, multiple-baseline single-case experimental design (SCED) will be used in this study. In a SCED study, participants are repeatedly measured during all phases. The current study will be divided into four phases: baseline, treatment, maintenance, and follow-up. Repeated and frequent measures of the target behavior will be conducted within each phase. The rule of thumb is to collect data at least three, preferably five times per phase [38,39]. The primary outcome measure will be assessed daily during the baseline phase and twice a week in the treatment and maintenance phase. All secondary outcome measures will be assessed at T0 (baseline); a subset will also be assessed at T1 (post-treatment) and T2 (follow-up; 3–5 months post-treatment). See Table 1 and Fig 1 for an overview of the phases and measurements included in this study, including both primary and secondary outcome measurements.

**Table 1:** Overview of the phases and measures T-ScEmo4ALL Plus^+^.

Tests and questionnaires	Description	T0	Baseline (A)	Treatment (B)	T1	Maintenance (C)	Follow-up (D)/T2
Phase duration (weeks)			2 up to 4	20 up to 25		13 up to 21	
**PATIENTS**							
***General information***							
Demographics	Sex, age, education level, marital status	▲					
Medical data	Type of brain injury, date and time since injury, injury severity, lesion location	▲					
***Social cognition***							
Social Functioning Scale (SFS)	Functioning in social situations	●	● (daily)	● (twice a week)		● (twice a week)	●
Brock’s Adaptive Functioning Questionnaire – social scales (BAFQ-SOC) [41,42]	Social monitoring, empathy	▲			▲		▲
Dysexecutive Questionnaire – social scales (DEX) [43]	Metacognition, social convention, behavioral emotional self-regulation	▲			▲		▲
Interpersonal Reactivity Index (IRI) [44]	Cognitive and affective components of empathy	▲			▲		▲
La Trobe Communication Questionnaire (LCQ) [45]	Communication quality	▲			▲		▲
Cartoon test [46]	Mental state attribution	▲			▲		▲
Facial expression of emotion: stimuli and tests (FEEST) [47]	Facial affect recognition	▲			▲		▲
***Neuropsychiatric symptoms***							
Neuropsychiatric Problems Scale (NPS)	Severity of neuropsychiatric (behavior) problems, e.g., irritability, aggression, depression, anxiety	▲	▲ (daily)	▲ (twice a week)		▲ (twice a week)	▲
***Partner relationship***							
Relationship Quality Scale (RQS)	Perceived quality of relationship with significant other	▲	▲ (daily)	▲ (twice a week)		▲ (twice a week)	▲
Dutch Marital Satisfaction and Communication Questionnaire (DMSCQ) [48]	Marital satisfaction, negative communication and open communication	▲			▲		▲
***Other cognitive measures***							
Trail Making Test (TMT) [49]		▲					
Dutch version of the Rey Auditory Verbal Learning Test (15 Words Test) [50]		▲					
Zoo Mapping Test of the Behavioural Assessment of the Dysexecutive Syndrome (BADS) [43]		▲					
**SIGNIFICANT OTHERS**							
***Social cognition***							
Social Functioning Scale (SFS)	Functioning in social situations	●	● (daily)	● (twice a week)		● (twice a week)	●
Brock’s Adaptive Functioning Questionnaire – social scales (BAFQ-SOC) [41,42]	Social monitoring, empathy	▲			▲		▲
Dysexecutive Questionnaire – social scales DEX [43]	Metacognition, social convention, behavioral emotional self-regulation	▲			▲		▲
La Trobe Communication Questionnaire (LCQ) [45]	Communication quality	▲			▲		▲
***Neuropsychiatric symptoms***							
Neuropsychiatric Problems Scale (NPS)	Severity of neuropsychiatric (behavior) problems, e.g., irritability, aggression, depression, anxiety	▲	▲ (daily)	▲ (twice a week)		▲ (twice a week)	▲
Neuropsychiatric Inventory Questionnaire (NPI-Q) [51]	Neuropsychiatric symptoms	▲			▲		▲
***Partner relationship***							
Relationship Quality Scale (RQS)	Perceived quality of relationship with significant other	▲	▲ (daily)	▲ (twice a week)		▲ (twice a week)	▲
Dutch Marital Satisfaction and Communication Questionnaire (DMSCQ) [48]	Marital satisfaction, negative communication and open communication	▲			▲		▲
***Other***							
Caregiver Strain Index (CSI) [52]	Caregiver strain	▲			▲		▲

● = Primary Outcome Measure.

▲ = Secondary Outcome Measures.

SCED methodologies employ strategies designed to address concerns about internal validity. Key elements to consider in the design include randomization, blinding, and monitoring treatment adherence. The Risk of Bias in N-of-1 Trials (RoBiNT) Scale will be used to monitor and report the methodological rigor (i.e., internal and external validity) of the study design [40].

#### Randomization.

The multiple baseline design will be used to strengthen internal validity by controlling for threats such as history and maturation (i.e., in the case of brain injury spontaneous or natural recovery should be considered). Three different baseline lengths will be used: start treatment within 16–20 days, 21–27 days, or 28–33 days. This setup ensures that no participant waits longer than 4 weeks before starting T-ScEmo treatment, which aligns with the average waiting period for non-participating patients. The participants are randomly assigned to a baseline length by using a randomization tool. Randomization applies only to baseline length; all participants receive the intervention.

#### Blinding.

Blinding participants and practitioners to phase is not feasible due to the behavioral nature of the intervention. Participants become aware of their baseline length after signing for study participation since they are told when the treatment starts (i.e., appointments are scheduled). The investigator is not blind since she coordinates and delivers the treatment. To reduce bias, the investigator will not visually or statistically analyze any data until data collection is complete. Additionally, neuropsychological assessments will be administered by trained staff (e.g., research assistants, interns or neuropsychologists) who are blinded to treatment phase through randomized scheduling. Participants complete digital questionnaires independently at home.

#### Therapist training and treatment adherence.

Given the complexity of social cognition problems and the presence of behavioral problems, the treatment is delivered exclusively by (clinical) neuropsychologists who engage in frequent peer consultation sessions to ensure consistent and high-quality application of the T-ScEmo protocol. Treatment adherence will be monitored using a checklist, where the therapist logs completed components and homework. Deviations or additions will be noted. Adherence percentages will be calculated based on session content and homework completion.

#### Ethics and dissemination.

The Ethics Review Committee Psychology and Neuroscience of Maastricht University provided ethical approval for the study (date 12-17-2024, reference number 289_121_12_2024). Additionally, the study protocol has been reviewed and approved by the institutional ethical committee (Commissie Wetenschappelijk Onderzoek) of GGZ Oost Brabant (date 12-24-2024, ref. 06.809/5315). All patients and their significant others will provide written informed consent prior to participation. Ethical and safety considerations are integrated throughout the study design, with particular attention to the inclusion of potentially vulnerable individuals with ABI and neuropsychiatric problems. Confidentiality of participant data will be ensured through secure data storage and pseudo-anonymization procedures (i.e., coding) with applicable data protection regulations.

The results of the study will be disseminated through peer-reviewed international publications, conference presentations, and stakeholder meetings. In addition, de-identified datasets and accompanying documentation will be made available in an open-access research data repository to facilitate transparency and replication. The study will be reported in accordance with the Single-Case Reporting guideline In Behavioural Interventions (SCRIBE; [53]), ensuring clarity and completeness in reporting.

### Participants

The goal is to include at least 8 patients receiving treatment at the Center of Excellence for Brain Injury and Neuropsychiatry (GGZ Oost Brabant), providing in- and outpatient care for patients with neuropsychiatric problems following ABI. The most common neurological diagnoses in this setting include stroke (ischemic, hemorrhagic, including subarachnoid hemorrhage), TBI, post-anoxic encephalopathy, brain tumors, and infections such as meningitis. Eligibility will be assessed for both clinical and outpatient populations.

Participants will be individuals with an established and documented history of ABI and current neuropsychiatric problems. These neuropsychiatric problems are broadly defined to include a wide range of behavioral problems, regardless of whether they meet formal DSM-5 criteria, that warrant referral and admission to our institution. As such, patients presenting with behavioral problems such as aggression, impulse control problems, disinhibited behavior, compulsion, and apathy are eligible for inclusion, even in the absence of a DSM-5 classification. In this study, patients present with neuropsychiatric problems of such severity and complexity that treatment within a specialized neuropsychiatric setting is indicated. These patients differ from those commonly seen in standard rehabilitation settings, who may also present with social cognition deficits, but exhibit less severe and disruptive neuropsychiatric symptoms.

For each participating patient, a significant other will also be included in the study. Ideally, this is the patient’s life partner, however, when no partner is available, a close family member (e.g., parent or sibling) or a close friend may fulfill this role. The significant other provides valuable information about the patient, their relationship, and their own experience related to the patient’s condition, and can also support the patient throughout the treatment.

#### In- and exclusion criteria.

The in- and exclusion criteria are largely consistent with those outlined in the T-ScEmo4ALL treatment protocol [54]. A key deviation, however, is that patients with comorbid neuropsychiatric problems are not excluded from participation. Accordingly, individuals with ABI and co-occurring neuropsychiatric problems are eligible for inclusion in this study. Inclusion criteria are presented in Table 2. This table is adapted from Heegers et al. [54].

**Table 2:** Overview of inclusion and exclusion criteria T-ScEmo4ALL Plus^+^.

Inclusion criteria patient	Inclusion criteria significant other	Exclusion criteria
Presence of social cognitive impairments, (i.e., impairments in emotion recognition, ToM, or social behaviour>1 of 4 tests indicative and/or>1 of the (sub)scales of relevant questionnaires indicative and/or, if available, the presence of frontal lesions on CT or MRI), judged by a neuropsychologist	Preferably a life partner. If a life partner is not available, a family member or close friend	Serious neurodegenerative conditions interfering with treatment
Presence of a neurological disorder (i.e., stroke, brain tumor, TBI, infections, post-anoxic encephalopathy)	Chosen by patient	Incapacity to make informed choices, to be judged by the neuropsychologist
Presence of current neuropsychiatric problems (i.e., aggression, impulse control problems, disinhibited behavior, compulsion, and apathy)	Age > 18	Serious cognitive disorders (aphasia, amnesia, disorders in visuoperception) interfering with treatment, to be judged by a neuropsychologist
Chronic state (6 months post-injury) to minimize the influence of spontaneous recovery		No significant other available who can fill out the questionnaires
Age between 18 and 70 years of age		Not able to speak and understand the Dutch language

### Intervention

T-ScEmo is an evidence-based, multifaceted treatment protocol designed to improve social cognition and emotion regulation in patients with ABI. T-ScEmo is part of standard clinical practice in the Netherlands and has been implemented at the Center of Excellence for Brain Injury and Neuropsychiatry since 2024, constituting ‘care as usual’. It is included in the Dutch guideline for neuropsychological rehabilitation [55], which recommends T-ScEmo as the preferred intervention for addressing deficits in social cognition. The T-ScEmo treatment is thus recommended by leading experts in clinical neuropsychology.

T-ScEmo treatment comprises 20 one-hour sessions and is structured into extended psychoeducation (for both patient and the significant other) followed by 3 core modules: emotion perception (module 1), perspective taking and understanding social information (module 2), and goal-directed social behavior (module 3). These modules are designed to be interdependent and mutually reinforcing, therefore, the developers strongly recommend delivering the full protocol. The presence of a significant other is required in the first psycho-education session and during the third module. Modules 1 and 2 follow a fixed structure across 10 sessions, while module 3 allows for tailoring based on individual needs and personal goals. Each session follows a consistent format: (1) review of homework assignments (5–10 minutes), (2) presentation and practice of new material, i.e., content of the session (45–50 minutes), and (3) preview of the next session with new homework assignments (5 minutes). Therapists familiar with the T-ScEmo are expected to require around 15 minutes of preparation time per session (printing documents, reviewing content). An overview of the treatment sessions, content and practical information is presented in Table 3. This table is adapted from Heegers et al. [54].

**Table 3:** Content of T-ScEmo protocol and practical information.

**T0 – BASELINE MEASUREMENTS**	Neuropsychological tests: face to face Questionnaires: digital Setting treatment goals
**PSYCHO-EDUCATION**	
Session 1**	Explanation of findings following the neuropsychological assessment and psycho-education
**MODULE 1**	**Perceiving social information**
Session 2	Perceiving emotional facial-features
Session 3	Facial-features and mimicry
Session 4	Facial-features and personal emotional experiences
Session 5	Perceiving and recognizing disapproval and shame
Session 6	Perceiving and recognizing corrective emotions, such as shame and disapproval/discomfort
**MODULE 2**	**Understanding social information**
Session 7	Thoughts, feelings, and behaviour of the other
Session 8	Perspective taking
Session 9	Empathy
Session 10	Complex social information: contradiction
Session 11*	Interim evaluation
**MODULE 3**	**Regulating behaviour in social situations**
*Note:*	*Module 3 includes several optional components. A total of 9 sessions may be devoted to Module 3. The selection of sessions is based on the participant’s goals*
Session 12	Personal space
Session 13*	Listening skills, turn-taking and timing
Session 14*	Checking emotions and thoughts of the other
Session 15	Training in social problem-solving
Session 16a	Understanding behaviour: registration
Session 16b	Social contacts: initiating and restoring contact
Session 17a	Understanding behaviour: registration of anger
Session 17b	Social contacts: appreciating & strengthening
Session 18*	“Inappropriate” behaviour and correcting feedback
Session 19a	Behaviour in stressful situations I (time-out + cue card)
Session 19b	Behaviour in stressful situations II (apologizing)
Session 20	Treatment evaluation
**T1 – POST-TREATMENT MEASUREMENTS**	Neuropsychological tests: face to face Questionnaires: digital
**T2 – FOLLOW-UP MEASUREMENTS**	Neuropsychological tests: face to face Questionnaires: digital

* Presence of a significant other is *desirable.*

** Presence of a significant other is *necessary.*

### Procedure

Healthcare professionals at the Center of Excellence for Brain Injury and Neuropsychiatry who identify patients with an indication for treatment of social cognition problems will inform the researcher. Recruitment started on 1 January 2025. Participant recruitment is ongoing and will continue until the end of December 2026. Completion of data collection is anticipated in April 2027, and the study results are expected to be available by December 2027.

Psychologists will screen patients for eligibility based on the in- and exclusion criteria. Eligible patients will receive detailed information about the study. Then, patients will be invited to participate voluntarily during outpatient or clinical appointments. Those who consent to participate will be asked to sign an informed consent form. Upon providing informed consent, participants will be randomized to a baseline phase of varying duration, after which the T-ScEmo intervention starts, followed by maintenance and follow up phases.

Baseline measurements include a neuropsychological assessment, unless one has been conducted within six months prior to the start of the study. Furthermore, during this initial session, the therapist, together with the patient and the significant other, will formulate three treatment goals (TGA; Treatment Goal Attainment). Before, during, and after treatment participants complete multiple neuropsychological tests during a face-to-face appointment with a (trained) psychological assistant, intern or neuropsychologist. Participants and the significant other will complete a series of questionnaires digitally (see Table 1). They are free to choose when to complete the questionnaires and may pause and resume as needed.

In phases B and C, self-report scales are administered twice a week and must be completed before the next treatment session to ensure timely data collection. Post-treatment measurements will be conducted to evaluate the maintenance of treatment effects and to maintain consistency in the number of measurements across participants. A follow-up measurement will be conducted 3–5 months after treatment completion to evaluate long-term efficacy. Patients who choose not to participate will still receive T-ScEmo treatment, as it is considered standard care.

### Primary outcome measure

*Satisfaction with social functioning* is assessed using a Social Functioning Scale (SFS), in which patients and significant others rate the patient’s functioning in social situations on a 10-point Likert scale ranging from 1 (“not at all”) to 10 (“entirely”). The item presented is: “How satisfied are you today with your (or your significant other’s) functioning in social situations?” To ensure a sufficient number of data points, as recommended for SCED studies, the SFS will be completed frequently: daily during phase A (2–4 weeks) and twice per week during phases B (20–25 weeks) and C (13–21 weeks). The SFS will be completed by both the patient and a significant other online (M-Path).

### Sample size

We aim to include at least 8 patients and their significant others (i.e., 16 participants in total), with a maximum of 20 patients and their significant others (i.e., up to 40 participants). According to Tate et al. (2019) [56], a minimum of 3 participants is generally recommended to ensure sufficient variability and reliability of the data, as well as meet evidence standards. Moreover, replication across at least 3 participants is essential to draw robust conclusions. Given that the study design consists of 3 different baseline lengths – each considered a separate experiment in terms of the SCED design standards – it is ideal to include at least 3 participants per 3 baseline condition (i.e., a total of 9 participants) [56].

### Data analysis

Descriptive analyses (count, percentage, median, and range) will be performed to describe sociodemographic (e.g., age, sex), clinical (i.e., types of ABI, time since ABI, DSM-5 classifications), and treatment characteristics (i.e., therapists involved, adjustments to standard treatment protocol, total number of sessions, dropout).

A wide variety of analytic techniques is available for evaluating data from SCED studies, including both visuals analysis and statistical methods.

#### Visual analyses.

As a first step, we will present the SCED data graphically and use visual inspection to determine whether differences between phases are apparent. The underlying assumption is that if differences are not visually apparent, they are unlikely to be statistically significant and clinically meaningful. Scores on frequently administered measures will be plotted graphically for each participant using the Shiny app for Single-Case Data Analysis (Shiny SCDA) in R [57,58] and analyzed visually. Intervention-related changes in outcome measures and the duration of change will be assessed based on the procedure described by Lane and Gast (2014) [59]. Visual analyses will focus on level, trend, variability, and overlap (i.e., extent of data overlap across phases). Evidence for a functional relationship will be categorized as follows: strong evidence (at least three demonstrations of the intervention effect and no demonstration of no effect), moderate evidence (at least three demonstrations of the intervention effect with at least one demonstration of no effect), and no evidence (fewer than three demonstrations of the intervention effect).

#### Statistical analyses.

Statistical analyses of SCED data have gained prominence in recent years and will be used as a complementary method of data evaluation. There are still, however, no agreed-upon criteria for statistical analysis of SCED data [60]. Data from the frequently administered measures will be displayed and analyzed using the Shiny app for Single-Case Data Analysis [57]. The generated graphs will support visual analysis, and regression analysis will be conducted for quantitative assessment. Randomization tests (RTcombiP; [61]) will be applied to evaluate if levels of social cognition problems, social communication, social interaction, social problems, and neuropsychiatric problems differ significantly between phases (baseline versus treatment + follow-up).

If visually analysis yields strong or moderate evidence, effect size estimates will be calculated using non-overlap of all pairs (NAP) for the dependent variables: social cognition problems, social interaction, social communication, neuropsychiatric problems, and neuropsychiatric problems. To assess individual improvements on the periodic measurements (BAFQ, DEX, IRI, FEEST, Cartoon test, LCQ, DMSCQ, NPI-Q), the Reliable Change Index (RCI) will be calculated. The RCI will indicate whether post-treatment and follow-up changes are both statistically and clinically significant.

Reporting of the study will follow the Single-Case Reporting guideline In Behavioral interventions (SCRIBE) checklist [53], consisting of 26 items that outline the structure of a SCED report.

## Discussion

To date, no study has examined the effectiveness of the T-ScEmo treatment in individuals with ABI and co-occurring neuropsychiatric problems. The current study protocol is the first to address this gap. Building on previous research in TBI populations [5,21], we hypothesize T-ScEmo will similarly improve social functioning in this more complex group as social cognition problems often stem from comparable neuropathological mechanisms. Furthermore, we anticipate that treatment gains will be sustained over time.

In addition to improvements in social functioning, we expect secondary benefits in neuropsychiatric symptomatology. Social cognition problems are linked to increased emotional distress and behavioral dysregulation; therefore, improvements in this domain may alleviate psychiatric burden [28]. However, neuropsychiatric problems such as reduced learning capacity, low motivation, and limited adaptability may also hinder treatment engagement and outcomes. The extent of such interference likely depends on the type and severity of neuropsychiatric problems. To explore this interaction, neuropsychiatric problems will be systematically assessed and monitored throughout the study. These data may inform future protocol adaptations aimed at personalizing treatment for specific symptom profiles. Moreover, individuals exhibiting complex behavior following ABI are increasingly encountered outside specialized neuropsychiatric settings, including within medical specialist rehabilitation settings. This trend underscores the growing clinical need for accessible, evidence-based interventions that are suitable for use across diverse neurorehabilitation contexts and responsive to the challenges posed by this expanding patient population.

The original T-ScEmo protocol is implemented without modification to allow for accurate assessment of its effectiveness in this patient population. However, challenges are anticipated. The combination of ABI and neuropsychiatric problems often result in complex clinical presentations. For example, cognitive or emotional dysregulation may necessitate shorter sessions and more flexible pacing. Reduced motivation may also negatively impact both adherence to treatment and response to assessments. These issues will be carefully monitored, documented, and reported accordingly.

Given the small and heterogeneous sample in this highly specialized clinical setting, an RCT was considered unfeasible. Instead, a single-case experimental design (SCED) was chosen, incorporating randomized baseline lengths to enhance internal validity. One challenge of the SCED approach is the intensive data collection schedule, including daily and biweekly measurements. This may reduce response rates due to motivational issues or limited illness insight. To mitigate this risk, adherence will be closely monitored by both the therapist and researcher on a weekly basis. For inpatients, nursing staff may support data collection, while the M-path app provides automatic notifications to encourage timely questionnaire completion. Multiple assessment points have been incorporated to ensure that sufficient data is collected even in the event of missing responses.

## Conclusion

This study aims to evaluate the effectiveness of the T-ScEmo protocol in individuals with ABI and comorbid neuropsychiatric problems. The T-ScEmo may offer a valuable addition to neurorehabilitation practices. Results may contribute to improved, more personalized treatment strategies for this underrepresented patient population.

## Supporting information

S1 File SPIRIT checklist. SPIRIT 2025 checklist of items to address in a randomized trial protocol.
(DOCX)
